# Single-Cell Quantification of mRNA Expression in The Human Brain

**DOI:** 10.1038/s41598-019-48787-w

**Published:** 2019-08-26

**Authors:** Sarah Jolly, Verena Lang, Viktor Hendrik Koelzer, Carlo Sala Frigerio, Lorenza Magno, Patricia C. Salinas, Paul Whiting, Ernest Palomer

**Affiliations:** 1 ARUK-UCL Drug Discovery Institute, University College London, London, United Kingdom; 2 Department of Pathology and Molecular Pathology, University and University Hospital Zurich, Zurich, Switzerland; 3 Department of Oncology and Nuffield Department of Medicine, University of Oxford, Oxford, United Kingdom; 4 UK Dementia Research Institute, University College London, London, United Kingdom; 5 Department of Cell and Developmental Biology, University College London, London, United Kingdom

**Keywords:** Transcription, Molecular neuroscience

## Abstract

RNA analysis at the cellular resolution in the human brain is challenging. Here, we describe an optimised approach for detecting single RNA transcripts in a cell-type specific manner in frozen human brain tissue using multiplexed fluorescent RNAscope probes. We developed a new robust analytical approach for RNAscope quantification. Our method shows that low RNA integrity does not significantly affect RNAscope signal, recapitulates bulk RNA analysis and provides spatial context to transcriptomic analysis of human post-mortem brain at single-cell resolution. In summary, our optimised method allows the usage of frozen human samples from brain banks to perform quantitative RNAscope analysis.

## Introduction

Molecular characterisation of different cell-types in complex human tissues such as the brain is of crucial importance for both fundamental and applied research. The human brain is composed of potentially hundreds of different cell types displaying complex morphologies and often long, ramified and entwined processes, making its dissociation into single cells a difficult process. RNA-seq of immunopanned human brain cell types has been performed from fresh biopsies obtained from neurosurgical site^1^, but these samples are non-existent for neurodegenerative diseases; instead, neuroscientists normally access frozen or fixed post-mortem human brain samples from brain banks. However, single-cell preparation from snap frozen tissue is extremely difficult, thus making single-cell sorting virtually impossible. To overcome these limitations, several laboratories have performed single-nucleus RNA-seq. This method can be readily applied to frozen tissue samples and it has been shown to well recapitulate the cellular transcriptome^2,3^. Nonetheless, this method has its own limitations, notably the loss of any compartmentalised cytoplasmic RNA^3^. Single-cell or single-nucleus-based transcriptomics fail to capture spatial context, cell-to-cell relationships and circuitry structure. *In situ* hybridisation (ISH), which visualises specific mRNA molecules at a cellular level, is ideal to study gene expression in different cell types because it uses intact tissue sections, therefore maintaining tissue integrity, cell structure and morphology^4,5^. Additionally, ISH has been successfully used for systematic analyses of RNA expression in the rodent brain (Allen Brain Atlas). Over the last decade, new and more sensitive ISH methods have been developed^4^, including single molecule RNA fluorescent *in situ* hybridization (smFISH)^6,7^, enabling quantification of RNA expression levels in specific cell types at single-cell resolution. Crucially, post-mortem human brain tissue presents higher rates of RNA degradation^8^, making bulk and single-cell or single-nuclei RNA analyses more complex.

Alzheimer’s disease (AD) is a devastating neurodegenerative disorder that accounts for two thirds of total dementia cases, and is characterised by progressive cognitive impairment and memory loss leading to difficulties in daily tasks^9^. The AD brain displays extracellular amyloid beta (Aβ) deposition and intracellular tau aggregates (neurofibrillary tangles), resulting in synapse loss and neuronal death^10^. In addition, AD has been associated with changes in the molecular profile of microglia and astrocytes^11,12^. Thus, studying molecular changes in human AD samples at single-cell resolution by a combination of smFISH and image analysis methods is critical for elucidating the mechanisms underlying neurodegenerative processes, including the investigation of cell-cell relationships and the impact of secondary features (e.g. Aβ plaques) in spatial context.

In this study, we show that multiplexed RNAscope, a commercially available smFISH assay^13^, can be successfully employed in frozen human hippocampal samples obtained from brain banks. In addition, we developed an analysis pipeline to quantify levels of single RNA molecules in major cell-type populations of the human brain with single-cell resolution. Importantly, we show that low RNA integrity score (RNA Quality Indicator; RQI = 2.9) does not significantly affect smFISH signal, thus frozen samples with partially degraded RNA could be used for smFISH. By analysing the expression of three cell-type specific genes (*SLC1A2* for astrocytes, *MAP2* for neurons and *P2RY12* for microglia), we demonstrate that our adapted smFISH protocol is suitable to discriminate RNA expression in a cell-type specific manner. Finally, we use our optimised smFISH protocol and pipeline analysis to study gene expression in the context of neurodegeneration. We performed multiplexed smFISH to detect three cell-type specific genes known to be up- or downregulated in AD: *TREM2* in microglia, *SNAP25* in neurons and *DKK1* in neurons and astrocytes. Our results for *TREM2*, *SNAP25* and *DKK1* compare favourably to bulk qPCR and recapitulate previously reported changes in expression between control and AD cases.

## Results

### RNAscope can be used in frozen human brain samples

**Figure 1:**
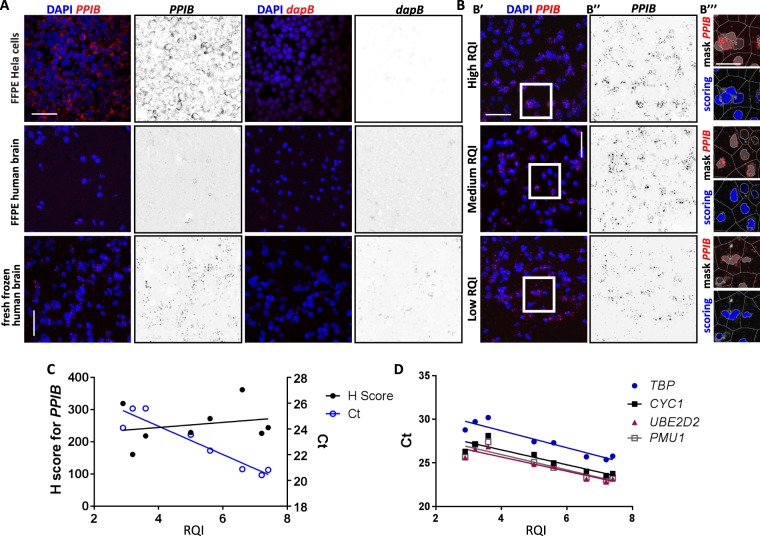
Use of RNAscope to visualise gene expression in human hippocampal biopsies from healthy individuals. (**A**) Confocal images of Hela cell FFPE pellets (top panel), FFPE (middle panel) and fresh frozen (bottom panel) hippocampal human sections showing no signal for the negative control (*dapB*) and variable signal levels of the positive control probe (*PPIB*; red). DAPI indicates nuclear staining (blue). Scale bars 50 µm. (**B**) Confocal fluorescent RNAscope shows expression of *PPIB* in fresh frozen hippocampal sections with high (7.2–7.4, top panel), medium (5–6.6, middle panel) and low (2.9–3.6, right panel) RNA Quality Indicator (RQI). B’ shows double staining for DAPI (blue) and *PPIB* (red). B” shows *PPIB* expression (black). (B’’’) Mark-up overlay illustrating the cell by cell segmentation and scoring of the ISH signals (nuclear colour coding indicates the expression level 0+ = black, increasingly brighter shades of blue indicating 1+, 2+, 3+ and 4+ cells). Scale bars 50 µm, zoom imaged 20 µm. (**C**) Black filled circles: quantification of the number of puncta/cell (H-score) in *PPIB*-positive cells showing no differences in sections with different RQIs (R = 0.227; p = 0.5889). Blue hollow circles: qPCR values (Ct) for *PPIB* gene showing a correlation between RQI and Ct (R = −0.942; p = 0.0004). (**D**) Quantitative qPCR showing a correlation between Ct and RQI of four housekeeping genes, including *PMU1* (R = −0.918; 0.0013), *TBP* (R = −0.941; p = 0.0004), *CYC1* (R = −0.916; p = 0.0013) and *UBE2D2* (R = −0.936; p = 0.0006).

The aims of this study were to establish a protocol for visualising specific mRNA species in different human brain cell types, and to develop a method to quantify the data. We used this technique on tissue samples from brain banks, which are preserved as formalin-fixed paraffin-embedded (FFPE) tissue blocks or fresh-frozen material. First, we performed smFISH in FFPE HeLa cells (Fig. 1A) and FFPE human hippocampal sections (Fig. 1A). Probes against the widely expressed Peptidylprolyl Isomerase B (*PPIB*) and against the bacterial gene *dapB* were used as positive and negative controls, respectively. Although a study successfully used chromogenic-RNAscope in human hippocampal sections^14^, our fluorescence read-out was not conclusive, with only a few cells showing expression of *PPIB*. Another study reported the use of chromogenic-BaseScope (an RNAscope approach using shorter probes) in human brain tissue^15^. We therefore performed BaseScope in FFPE human hippocampal sections but we could not detect any consistent signal (data not shown), and the fact that BaseScope cannot be multiplexed limits the applicability of this method. Based on these results, we hypothesised that long formalin fixation (2–3 weeks) and/or deparaffinisation might degrade RNA or block accessibility of the probes to the target RNA, thus impairing smFISH staining.

To overcome the limitations of long-term fixation of FFPE protocols used by brain banks, we hypothesised that smFISH could be successfully implemented by using frozen tissue with high RNA integrity. We extracted RNA from different hippocampal samples and analysed RNA integrity by determining the RNA Quality Indicator (RQI) score (from 1 to 10, with 10 being the best quality). RQI of our samples ranged from 2.9 to 7.4. Samples with high RQI (above 7) were selected to test whether human hippocampal sections from frozen samples can be used for smFISH assays. A clear signal for the positive probe (*PPIB*) was detected in comparison with the negative control probe (*dapB*; Fig. 1A). These experiments demonstrate that RNAscope can be successfully used in frozen human brain samples with high RNA integrity.

### RNA integrity does not affect RNAscope signal

The brain is characterised by a higher RNA degradation rate than other tissues^8^. It has been shown that low RNA quality (RQI < 3.9) negatively impacts on qPCR amplification^8^ and is not optimal for RNA-seq^16^. Samples with low RQIs must therefore be excluded from the experimental datasets. To determine if low RQI influences RNAscope signal, we performed smFISH on frozen brain tissues from three different RQI groups: high (7.2–7.4), medium (5–6.6) and low (2.9–3.6; Fig. 1B’,B”). We did not observe qualitative differences in smFISH puncta per cell between the different RQI groups. However, to conclude if low RQI impacts on smFISH signal, proper quantification of fluorescent puncta and their correct attribution to the cognate cell is paramount.

To quantify smFISH puncta, we have implemented a new approach using the HALO v2.3.2089.18 (Indica Labs, Corrales, NM, USA) software platform. This approach allows reproducible quantification of mRNA signals in multiplexed smFISH at single-cell resolution and provides spatial coding and morphometric characterization of the cell populations of interest (see Methods for details). Briefly, we performed colour deconvolution to optimise the detection of probes and nuclear counterstain. We optimised nuclear segmentation and contour detection using the colour and intensity attributes of the nuclear counterstain in combination with cell classification features. Absolute cell counts were derived for each region analysed. Probe detection was performed based on channel deconvolution and probe signals were assigned to each nucleus by proximity. Cell-by-cell smFISH signal counts were derived for each probe on a continuous scale. Graphical mark-ups were generated to visualize cell-by-cell probe quantification within the microarchitecture of the hippocampus (Fig. 1B’’’ and Supp. Fig. 1, see Methods for further detail). By using this new quantitative approach, we analysed *PPIB* levels (Fig. 1C) and observed that low RQI is not associated with a reduced number of smFISH puncta per cell (H-score; Fig. 1C black filled dots, R = 0.227, p = 0.5889). In contrast, RQI affected qPCR amplification of *PPIB* and other reference genes in the same samples (Fig. 1C,D blue hollow dots, R = −0.942, p = 0.0004), as previously reported^8^. To further demonstrate that low RQI does not significantly impact on the count of smFISH signals, we quantified the levels of another common housekeeping gene (TATA-Box Binding Protein; *TBP*) in samples covering a range of RQI, from low to high. As shown for *PPIB*, low RNA integrity does not result in different levels of smFISH signal for *TBP* (Supp. Fig. 2). Altogether, these data indicate that RNAscope can be successfully employed in frozen human brain sections even with low RNA integrity (RQI ≥ 2.9).

### Cell-type discrimination of differentially expressed genes by RNAscope

**Figure 2:**
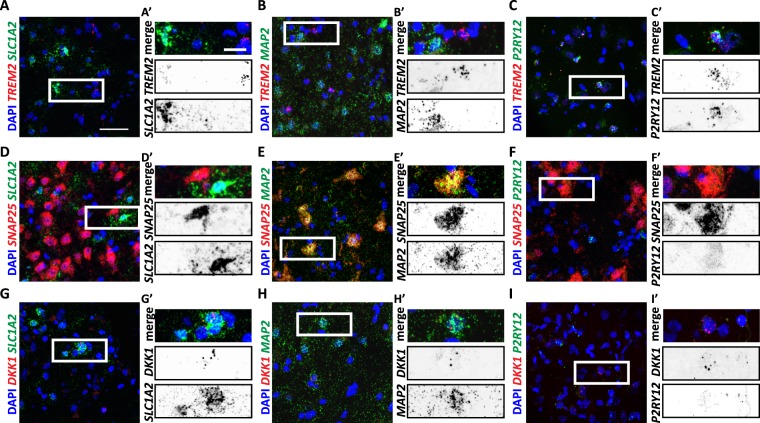
Single-cell resolution of *TREM2*, *SNAP25* and *DKK1* mRNAs present differential cell-type expression. Confocal images of control hippocampal slices showing *TREM2*, *SNAP25* and *DKK1* co-localising with different cell-type-specific markers including *SLC1A2* (astrocytic), *MAP2* (neuronal) and *P2RY12* (microglial). (**A**–**C**) RNAscope of *TREM2* (red) showing no colocalization with *SLC1A2* (A-A’; green) nor *MAP2* (B-B’; green), but *P2RY12* (C-C’; green). (**D**–**F**) *In situ* hybridisation of *SNAP25* (red) showing no colocalization with *SLC1A2* (D-D’; green) or *P2RY12* (F-F’; green), but *MAP2* (E-E’, green) (**G–I**) RNAscope of *DKK1* (red) showing colocalization with *SLC1A2* (G-G’; green) and *MAP2* (H-H’, green) but not with *P2RY12* (I-I’; green). DAPI indicates nuclear staining (blue) in all cases. Scale bars 50 µm, zoom imaged 20 µm.

The secondary aim of this study was to establish a multiplexed smFISH protocol to study changes in RNA levels in a cell-type specific manner in the human brain. Therefore, we next demonstrated smFISH signal in three major cell types in the brain. Astrocytes, neurons and microglia were detected using cell-type specific probes against *SLC1A2*, *MAP2* and *P2RY12*, respectively (Fig. 2)^1^. We have also tested alternative probes for astrocytes (*SLC1A3*), neurons (*RBFOX3*) and microglia (*ITGAM* and *CST7*; data not shown). The evaluation of probe suitability was based on puncta distribution and levels of expression. The astrocytic probe against *SLC1A3* and the neuronal probe against *RBFOX3* are manly localised in the cytoplasmic compartment, making its attribution to a particular nucleus more difficult. The cytoplasmic localization of *SLC1A3* smFISH puncta is supported by the work of Bayraktar and collegues^17^. In addition, *SLC1A3* is also detected in some microglia^1^. Our results also show that *ITGAM* and *CST7* are less expressed than our primary microglial probe candidate, *P2RY12*. Indeed, single microglia RNA-seq show that *ITGAM* and *CST7* are less expressed than *P2RY12*^18^. Therefore, we suggest the use of *SLC1A2*, *MAP2* and *P2RY12* probes for labelling the above cell types in human frozen brain sections.

Subsequently, we used smFISH to determine the cell-type distribution of mRNA transcripts of three genes that are relevant to neurodegeneration: Triggering Receptor Expressed on Myeloid cells 2 (*TREM2*), Synaptosome Associated Protein 25 (*SNAP25*) and Dickkopf 1 (*DKK1*). *TREM2* is mainly expressed in microglia cells^1^ and polymorphisms in this gene are associated with AD^19^ and other neurodegenerative diseases. *SNAP25*, which is expressed in neurons and is essential at synapses, is decreased in AD^20^. *DKK1*, a secreted Wnt signalling antagonist, is expressed in neurons and astrocytes^1^, is upregulated in AD^21^, and mediates Aβ-induced synapse loss^22^. These genes are expressed at different levels, with *SNAP25* being highly expressed and *DKK1* expressed at low levels in healthy individuals^1^. Therefore, these three genes allow us to further analyse our smFISH staining at a wide range of gene expression levels. Consistent with previous reports, in control hippocampal samples, *TREM2* expression is restricted to microglia (Fig. 2A–C)^1^, *SNAP25* to neurons (Fig. 2D–F)^1^, and *DKK1* to neurons and astrocytes (Fig. 2G–I)^1^, as shown by colocalization with the cell-type specific probes. As expected, we observed differential expression levels of these three genes: *SNAP25* is abundantly expressed, whereas *DKK1* is sparsely expressed. Based on these data, we conclude that the use of frozen brain tissue for RNAscope is a sensitive and robust method for the visualisation of mRNA transcripts.

### Differential cell-type RNA expression in Alzheimer’s disease

**Figure 3:**
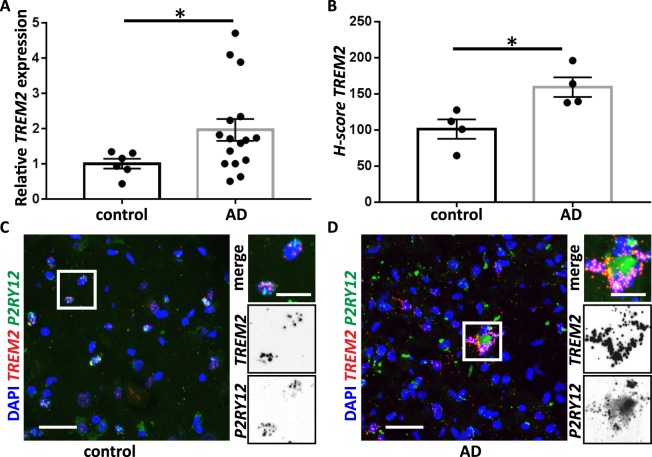
Microglia-specific upregulation of *TREM2* in fresh frozen human AD biopsies. (**A**) Bulk *TREM2* mRNA expression in human hippocampal samples analysed by qPCR showed a 1.96 fold increase of *TREM2* mRNA in AD (n = 16) vs. control (n = 6) samples. (*Indicates p = 0.045 by Mann-Whitney U Test). (**B**) Quantification of the number of dots/cell (H-score) in *TREM2*-positive cells for control (**C**; n = 4) and AD (**D**; n = 4) samples (*Indicates p = 0.0286 by Mann-Whitney U Test). (**C**,**D**) Representative confocal images of *TREM2* mRNA expression in control (**C**) and AD (**D**) samples. *TREM2* is shown in red, *P2RY12* in green. DAPI indicates nuclear staining (blue) in all confocal images. Scale bars 50 µm, zoom images 20 µm. Graphs showing mean value ± SEM.

Finally, to further test our modified RNAscope protocol and analysis pipeline, we compared the expression of *TREM2*, *SNAP25* and *DKK1* in hippocampal samples from 4 control and 4 AD tissue samples (RQI 3.9–7.4). This analysis was performed using the same pipeline described above (Supp. Fig. 1): signal counts for the cell-lineage specific genes, *MAP2*, *SLC1A2* and *P2RY12*, were used for cell-type identification and test probes (*TREM2*, *SNAP25* or *DKK1*) were analysed in particular cell types. qPCR analyses showed that *TREM2* expression is elevated in AD brains (Fig. 3A)^23^. Consistently, H-score quantification of smFISH signal showed upregulation of *TREM2* expression in AD samples (Fig. 3B–D). Note that in AD samples, the green channel shows a high background signal, even when using the autofluorescence quencher TrueBlack (Supp. Fig. 3). This high background level might be due to the presence of large protein aggregates in AD brain tissue. Therefore, this channel is not ideal for analyses of samples from patients with diseases presenting autofluorescent aggregates^24,25^. Bulk qPCR analyses revealed that *SNAP25* expression is decreased in AD^20^ (Supp. Fig. 4A). smFISH also showed a decrease in *SNAP25* (Supp. Fig. 4B–D), accompanied by reduced *MAP2* expression. However, *SNAP25* could not be quantified using the same method used for *TREM2*, as single RNA puncta could not be detected. Thus, for highly expressed genes we propose quantification of total smFISH intensity per positive cell. Finally, we analysed *DKK1* expression. Bulk qPCR confirmed an increased expression of *DKK1* in the hippocampus of AD patients^21^ (Supp. Fig. 5). Although the *DKK1* smFISH signal was above background, we could not quantify it because very few cells express *DKK1*. Altogether, these data indicate that our optimised smFISH protocol and analysis pipeline can be used to analyse cell-type specific changes in RNA levels in human frozen tissues from control and neurodegenerative cases at the single-cell resolution.

## Discussion

Our study describes a multiplexed smFISH methodology optimised to enable detection of RNA molecules at single-cell resolution in frozen human brain tissue. In addition, we describe an analysis pipeline that enables the quantification of multiplexed smFISH data at the single-cell level, allowing visualisation of single RNA transcripts and cell-type discrimination. Importantly, we demonstrate that RNA integrity is not a critical factor for using smFISH in frozen human samples. Together, this approach has widespread utility for the quantification of gene expression in complex human tissues at single-cell resolution, thus complementing large scale unbiased genomic approaches such as single-cell and single-nucleus RNA-seq.

A recent study showed that fresh human brain biopsies could be utilised for multiplexed smFISH^17^. These samples were taken from the neurosurgical site and processed for smFISH following standard PFA fixation, sucrose placement and OCT mounting. However, research laboratories outside clinical environments have limited access to samples of this nature. In addition, fresh biopsies from neurodegenerative cases are virtually non-existent. Instead, laboratories obtain human samples from brain banks, which generally provide FFPE or frozen samples. Although positive results can be achieved by chromogenic-RNAscope and BaseScope in human FFPE brain sections^14^, or by smFISH in human FFPE preserved gut^26,27^ or lymphoma^28^ tissues, there are no studies reporting multiplexed smFISH in human FFPE brain samples. Indeed, our results show that FFPE human brain sections are not suitable for fluorescent RNAscope (Fig. 1). In contrast, we show that smFISH can be successfully used to analyse sections of frozen human brain tissue. A previous report also showed that smFISH can be deployed in human cortex^29^. However, the smFISH staining seems weaker than the ones presented in this study. These differences may arise from section thickness, the fixation method or other steps of RNAscope staining and imaging, which are not clearly explained in the text. Therefore, our optimised RNAscope approach could potentially be used by any laboratory as frozen human brain samples are accessible through brain banks and we provide a detailed and replicable methodological section.

RNA integrity is a critical issue when analysing gene expression by qPCR or sequencing as fragmented RNA impairs qPCR amplification^8^ and also interferes with RNA-seq analyses^16^. However, how RNA integrity impacts on RNAscope staining had not been previously investigated. Our results show that low-quality RNA (RQI = 2.9) does not significantly affect smFISH detection of two housekeeping genes (*PPIB* and *TBP*), the microglial gene *TREM2* or the neuronal gene *SNAP25* (Fig. 1 and Supp. Fig. 2). A possible explanation for this difference between smFISH and qPCR sensitivity to low RQI RNA is that qPCR requires the intact cDNA sequence for amplification (from the 5′ of the forward primer to the 3′ end of the reverse primer), whereas RNAscope requires the annealing of only 3 pairs of the 20 possible double Z probes to produce a detectable signal. Therefore, shorter, partially degraded RNA molecules could still be detected using RNAscope probes, positioning smFISH for the analysis of human frozen samples with variable RNA integrity. Note that our human sample set presented a large range of RQI scores (from 2.9 to 7.4), of which 51.8% present an RQI score lower than 3.9. Therefore, more than half of our sample set would need to be excluded for qPCR experiments. However, studies on RNA expression could be performed on the entire sample set across a wide range of RQI values with our optimised protocol for RNAscope in frozen human samples. This approach could be used in any given collection of frozen human samples, strongly increasing the number of samples available for *in situ* transcriptomics analysis.

To prove that RNA integrity has no impact on smFISH, and to analyse genes related to neurodegeneration in a cell-type manner, we have established a digital pathology analysis pipeline using the HALO software. This approach uses single-cell segmentation to properly attribute smFISH puncta to cognate cells. Correct ascription of RNA molecules is paramount for obtaining a reliable H-score (mRNA expression levels per cell), in particular for multiplexed smFISH aiming to analyse RNA levels in a cell-type specific manner. A scarcity of methods has previously limited the quantification of RNA expression by smFISH. Most studies in the rodent brain used smFISH to determine if a particular cell-type expresses a given gene^30–32^, to quantify changes in smFISH as dots per area of the brain^33^ or used semi-quantitative scoring^34^. In the human brain only one other pipeline analysis using Harmony in the Perkin-Elmer workflow has been previously reported^17^. We have used HALO because analysis of smFISH puncta is possible regardless of the input imaging platform, making our method broadly applicable to different research settings. An important requirement for determining the H-score of an individual probe is the capacity to detect and quantify single smFISH puncta, as successfully demonstrated in this study for a combination of six genes relevant for lineage determination, AD pathogenesis and housekeeping in the human brain. The H-score of highly expressed genes, like *SNAP25*, cannot be calculated because single RNA molecules are indistinguishable. Therefore, when studying highly expressed genes we suggest analysing overall RNAscope intensity per cell. In addition, super-resolution microscopy might allow detection of a discrete smFISH signal, facilitating H-score quantification with the analytical pipeline described here. Quantification of genes expressed at very low levels also presents limitations. In this report we have shown that *DKK1* is expressed by very sparsely distributed cells, indicated by the absence of puncta in most fields of view. This pattern of expression is very likely to introduce a selection bias by the operator when acquiring the images. To overcome this potential issue, and to increase the analysis throughput of RNAscope, we suggest performing whole slide imaging with scanning fluorescence microscopes, coupled with our image analysis pipeline for quantification. When applied to multiplexed staining, this approach would allow systematic screening for the expression of different genes across large tissue samples.

Our analysis pipeline assigns all probe signals to the nearest nucleus by proximity within a maximum radius of 25 µm, approximately corresponding to the maximum diameter of the soma of the largest cortical neuron^35^. As shown in Supp. Fig. 1, cell borders are extended outwards from the geometric center of each nucleus to the nearest adjacent cellular border. smFISH puncta that are not associated with cell nuclei on the image plane are excluded for cell classification and scoring. In the absence of reference markers to determine cellular boundaries, these settings represent a *bona fide* approximation of the cellular arrangement in coherent tissue where one cell physiologically borders the adjacent. In areas presenting high cellular density, this approach models the increasing spatial compression, whereas in areas with low cellular density a maximum radius of 25 µm from the centre of the nucleus is accepted, which approximates the largest physiological extension of a cell soma of a neuron *in situ*^35^. Limitations in the use of this analysis include sectioning artefacts that can lead to inclusions/exclusion of smFISH puncta. On one hand, smFISH puncta located in cellular processes coming from cells outside the image plane could be included in the quantification. On the other hand, smFISH puncta located in cellular processes further than 25 µm away from the centre of the nucleus would be excluded from the quantification. Stochastically, these are rare events, as we observed the highest signal density in direct proximity to the corresponding nuclei (Fig. 2). Cell isolation and culture techniques are likely to provide the most accurate results when analysing smFISH signals located in dendrites, axonal and other cellular processes. To optimally capture cell morphology in culture, we expect that adaptation of the present protocol to include cytoplasmic or membranous staining for cell segmentation will be required.

Last, we used our optimised smFISH protocol and pipeline analysis to study differential RNA expression in AD. Our RNAscope results compare favourably to bulk qPCR experiments and recapitulate previously reported changes in genes associated with neurodegeneration. In this type of study, it is important to have age- and gender-matched control and AD groups, but this is often limited by the available samples. In our case, younger samples are overrepresented in the control group (Supp. Table 1). To test if age has an impact on the results presented in this study, we analysed the correlation between gene expression and age at death. Our results show that the expression of *DKK1*, *SNAP25* and *TREM2* is not correlated to the age of death in our control sample set (Supp. Fig. 6). For the AD group, our results show no correlation between age at death and gene expression for *DKK1*, *SNAP25* or *TREM2* when analysed by qPCR, nor for *TREM2* when analysed by smFISH. However, the age of the AD patient is correlated with lower expression of *SNAP25* (Supp. Fig. 6). This result is in line with previous reports showing that older AD patients exhibit greater neuronal loss in the hippocampus^36^. All together, these results show that our optimised approach is a sensitive and robust method to analyse changes in RNA levels by multiplexed RNAscope, opening the possibility to validate transcriptomics data at the single cell-type level in the intact tissue. This validation step will be important to support conclusions from single-cell and/or single-nuclei RNA-seq, such as the hypothesised subtypes of the major brain cell types^37^ or that perturbed molecular profiles of different brain cell types could be involved in distinct processes in neurodegenerative diseases, such as AD^18,37^.

In summary, we show that the multiplexed fluorescent RNAscope assay can be successfully used in frozen human brain tissue to analyse single RNA molecules in a cell-type specific manner at the single-cell resolution. In addition, we provide a new pipeline analysis for smFISH quantification with broad applicability to human tissue samples and increased methodological robustness permitting the use of samples with a wide range of RNA integrity values. These results broaden the opportunity to use human frozen tissue samples from brain banks and provide new opportunities for research on cell-type specific RNA expression in neurodegenerative diseases.

## Methods

### Human tissue

Anonymised human samples from control and AD subjects were obtained from Cambridge Brain Bank (CBB), Division of the Human Research Tissue Bank, Addenbrooke’s Hospital, Cambridge UK. All samples were obtained with informed consent under CBB license (NRES 10/HO308/56) approved by the NHS Research Ethic Services. Tissue was either stored at −80 °C or fixed in 10% neutral buffered formalin for at least 2 weeks before being embedded in paraffin. All experiments were performed in accordance with relevant guidelines and regulations. Demographic data, Braak stages, RQI and post-mortem intervals for each sample set are shown in Supp. Table 1.

### RNAscope *in-situ* hybridization

To detect single mRNA molecules, RNAscope was performed on frozen vs FFPE-fixed control and AD hippocampal slices. 15μm sections were cut from frozen biopsies, dried for 10 min at 40 °C and kept at −80 °C. 9 µm sections were cut from FFPE blocks. In this study, one positive (Homo sapiens *PPIB*), one negative (*Escherichia coli DapB*) control probe and 6 different probes against genes of interest were used (Supp. Table 2). *In situ* hybridization (ISH) was performed according to the protocol of the RNAscope Multiplex Fluorescent Reagent Kit v2 (Cat. No. 320293).

Briefly, slides from frozen brains were dried in the oven at 40 °C for 3–4 min prior to incubation in cold 4% PFA for 25 min. Slides were then dehydrated in 50%, 70% and 100% ethanol for 5 min each at room temperature (RT). After drying the slides for 5 min at RT, H_2_O_2_ was added for 10 min at RT. For antigen accessibility, slides were treated with Protease IV for 20 min at RT. For FFPE-fixed sections and HeLa cells, slides were baked at 60 °C for 1 hour before being deparaffinised in xylene and 100% ethanol. After drying the slides 5 min at RT, H_2_O_2_ was added for 10 min at RT. For antigen accessibility, slides were incubated in boiling antigen retrieval solution (<98 °C) for 15 min, washed in water twice, dehydrated in 100% ethanol and finally treated with Protease Plus for 40 min at 40 °C.

Frozen and FFPE-fixed sections were washed twice in PBS. C2 probes were diluted in C1 probes at a 1:50 ratio and incubated on the slides for 2hrs at 40 °C. C1 probes were detected with TSA-Cy3 (Perkin Elmer, REF FP1170) and C2 probes with TSA-Fluorescein or TSA-Cy5 (Perkin Elmer, REF FP1168). To quench the autofluorescence due to the accumulation of lipofuscin or other protein aggregates, slides were incubated with TrueBlack (Biotium, 23007) for 30 sec at RT. Before mounting the slices, DAPI (Perkin Elmer, REF 323108) was added to label the nuclei. A one-day protocol has been used in all experiments to preserve the quality of the slices.

### Image acquisition and pre-processing

Images were acquired using a Zeiss 880 confocal microscope (x40 objective, 1.3 NA). Settings were established during the first acquisition and not modified afterwards. Stack images of 10 steps with a 0.5 µm interval were used. Two representative sections per samples were selected and 3–4 regions of interest were imaged at 1024 × 1024 pixels for image analysis. All images were pre-processed using ImageJ (maximum z-projection).

### Image analysis

#### Quantification of *MAP2*, *P2RY12*, *PPIB*, *SLC1A2*, *TBP* and *TREM2*

All images were reviewed by a board-certified pathologist and expert in image analysis (VHK). Regions with technical artefacts or unspecific background staining were annotated in.xml overlays and excluded from analyses. We then developed an image analysis approach for scoring multiplex mRNA smFISH of the lineage specific probes *SLC1A2* (astrocytes), *MAP2* (neurons) and *P2RY12* (microglia) and the genes of interest *PPIB*, *TBP* and *TREM2* in brain tissue using the HALO smFISH quantification for multiple probes v1.1 module. Briefly, colour deconvolution was performed and excitation channels were assigned for detection of nuclear counterstains and mRNA probes (DAPI, nuclear, 405 nm blue channel; C1 probe, 568 nm, red channel; C2 probe, 488 nm or 647 nm, green channel). Nuclear segmentation and contour detection were optimised using the DAPI counterstain and pathologist-supervised cell classification features (nuclear size, roundness, nuclear intensity, nuclear contrast threshold and segmentation aggressiveness) in real-time tuning mode using graphical overlays. Cell nuclei and X-Y location coordinates were recorded and nuclear morphometric parameters were quantified for each cell. Probe detection was optimised based on signal size (minimum signal size 0.45 µm^2^), intensity of positive probe pixels and contrast threshold parameter settings. Probe signals were recorded both as number of individual fluorescent spots as well as total signal area in µm^2^ per cell on a continuous scale with conservative settings for spot segmentation and cluster counting (probe copy intensity threshold = 0.15, spot segmentation aggressiveness = 0.5). For absolute quantification of probe signals, physically separated puncta were always counted as one probe copy. A spot was classified as a cluster when its intensity surpassed the set probe copy intensity threshold. The probe count of each cluster is derived from absolute intensity divided by the copy intensity parameter. Thresholding for the copy intensity parameter was performed using individual probe signals in lineage-positive cells as internal positive controls taking into account any background. Probe signals were assigned to each nucleus by proximity within a maximum cell radius of 25 µm. Total counts for the lineage specific probes, *P2RY12*, *SLC1A2 and MAP2*, were used to determine cell identity (neurons, astrocytes and microglia). We considered a cell positive for a particular linage when presenting 2 or more counts. Signal counts for the test probes *PPIB*, TBP and TREM2 were quantified for each cell and were used for the classification of each cell as negative (0 copies), 1 + (Minimum Copies/Cell: 1), 2 + (Minimum Copies/Cell: 4); 3 + (Minimum Copies/Cell: 10); or 4 + (Minimum Copies/Cell: 16), as shown in Fig. 1B and Supp. Fig. 1. The total number of probe counts associated with a cell give it the number used when generating the H-score for the entire area analysed. H-scores were calculated as follows: H-Score = (1 × % Probe 1 + Cells) + (2 × % Probe 2 + Cells) + (3 × % Probe 3 + Cells) + (4 × % Probe 4 + Cells), following recommendations by Cell Diagnostics USA (Newark, CA, USA) for the evaluation of RNAscope assays. Final scores derived by this metric will have a range between 0 and 400. A file containing the pipeline analysis ready to use in HALO can be sent upon request.

#### Quantification *of SNAP25*

Due to high levels of expression, *SNAP25* gene expression was further measured as total intensity per cell *SNAP25* staining was quantified using ImageJ: overall intensity of the *SNAP25* signal was measured in arbitrary units and was normalised to the number of neurons counted per image (Supp. Fig. 4).

### RNA extraction and RNA integrity analysis

Total RNA from frozen hippocampal tissue was extracted with TRIzol Reagent (Thermo Fisher Scientific ref: 15596018) and DirectZol Miniprep RNA Kit (Zymo Research ref: R2052). Briefly, 20–50 mg of tissue was homogenised in 800 µL of TRIzol using pellet pestles (Sigma-Aldrich ref: Z359947) coupled to pellet pestles cordless motor (Sigma-Aldrich ref: Z359971) in 1.5 ml tubes. Once homogenised, samples were centrifuged for 5 min at 16,000 g at 4 °C to remove any remaining tissue pieces and the supernatant was transferred to clean 1.5 mL tubes. 200 µL of chloroform were added, tubes were vortexed for 5 sec and left at RT for 10 min. To obtain the phase separation, samples were centrifuged for 15 min at 10,000 g at 4 °C. The aqueous upper face containing the RNA was transferred into a clean tube and RNA was extracted with DirectZol Miniprep RNA Kit following manufacturer’s instructions and including DNase in column treatment. RNA was quantified by measuring absorbance at 260 nm using a Nanodrop ND-100 (Thermo Fisher Scientific). RNA integrity (RNA Quality Indicator; RQI) was analysed using RNA HighSense Assay chips (Bio-Rad ref: 7007155) and run in Experion™ Automated Electrophoresis Station (Bio-Rad). Briefly, samples were diluted to 1–5 ng/µL, loaded into RNA HighSense Assay chips and run. 18S and 28S peaks were detected and RQI was determined. Only samples with a RQI 3.9 or above were used for relative qPCR comparing control and AD cases^38^.

### Retrotranscription and qPCR

Retrotranscription to first strand cDNA was performed using RevertAid H Minus First Strand cDNA Synthesis kit (Themo Fisher Scientific ref: K1632). Briefly, 2000 ng of total RNA was used for cDNA synthesis following manufacturer’s instructions. 5 ng of original RNA was used to perform fast qPCR using GoTaq qPCR Master Mix (Promega ref: A6002) in a LigherCycler 480 (Roche) with the manufacturer’s protocol (Supp. Table 3). Primers were designed using OligoPerfect design (Thermo Fisher Scientific) and validated using *in silico* PCR (UCSC genome Browser) and Ensembl BLAST (Ensemble.org). Primers were purchased from Sigma-Aldrich (Supp. Table 4) and used at 0.5 μM final concentration. Finally, primer efficiency was tested using serial dilutions from a mixed pool of cDNAs from all the samples. All primers used showed an efficiency of 1.9 or above (Supp. Table 4).

### Reference genes selection for qPCR

21 possible reference genes were tested (Supp. Table 4). GeNorm from GenEx package was used to select the best combination of reference genes^39^. Briefly, intra- and inter-group (Control and AD) variability was calculated. A cut-off of 0.5 was applied for intra-group and a cut-off of 0.2 was applied for inter-group variances (Supp. Table 5). The leftover genes were utilised to calculate SD for each gene regardless of groups and associated SD when pooling reference genes (Supp. Fig. 7). Based on these results, 4 reference genes were selected to perform relative qPCR (*PMU1*, *TBP*, *CYC1* and *UBE2D2*).

### Statistics

All statistical analyses were performed using SPSS 25 (IBM). Outliers were assessed by box plot and extreme outliers (1.5 x interquartile ranges (IQR) above the third or below the first quartile) were removed from the dataset. Pearson correlation (R) was tested with a bivariate correlation tool. Exact p-values are indicated. qPCR and RNAscope comparisons between control and AD cases were analysed by Mann-Whitney U test. In the figures, asterisks indicate p-values (*p < 0.05).

## Supplementary information

Supplementaly information

## Data Availability

Data generated for this study and a file containing the pipeline analysis ready to use in HALO re available upon request.
