# Supplementary material for: Single-Cell Quantification of mRNA Expression in The Human Brain
Source: Sci Rep. 2019 Aug 26;9:12353. doi: 10.1038/s41598-019-48787-w (PMC6710275; doi:10.1038/s41598-019-48787-w)
Supplement: Supplementary file 1 — Supplementaly information [file 41598_2019_48787_MOESM1_ESM.pdf]

Single-Cell Quantification of mRNA Expression in The Human Brain

Sarah Jolly[1*], Verena Lang[1*], Viktor Hendrik Koelzer[2,3*], Carlo Sala Frigerio[4], Lorenza Magno[1], Patricia C. Salinas[5], Paul Whiting[1,4], Ernest Palomer[5#]

[1] ARUK-UCL Drug Discovery Institute, University College London, London, United Kingdom
[2] Department of Pathology and Molecular Pathology, University and University Hospital Zurich, Zurich, Switzerland
[3] Nuffield Department of Medicine, University of Oxford, Oxford, United Kingdom
[4] UK Dementia Research Institute, University College London, London, United Kingdom
[5] Department of Cell and Developmental Biology, University College London, London, United Kingdom
[*] Co-first authors
[#] Lead contact: e.palomer@ucl.ac.uk

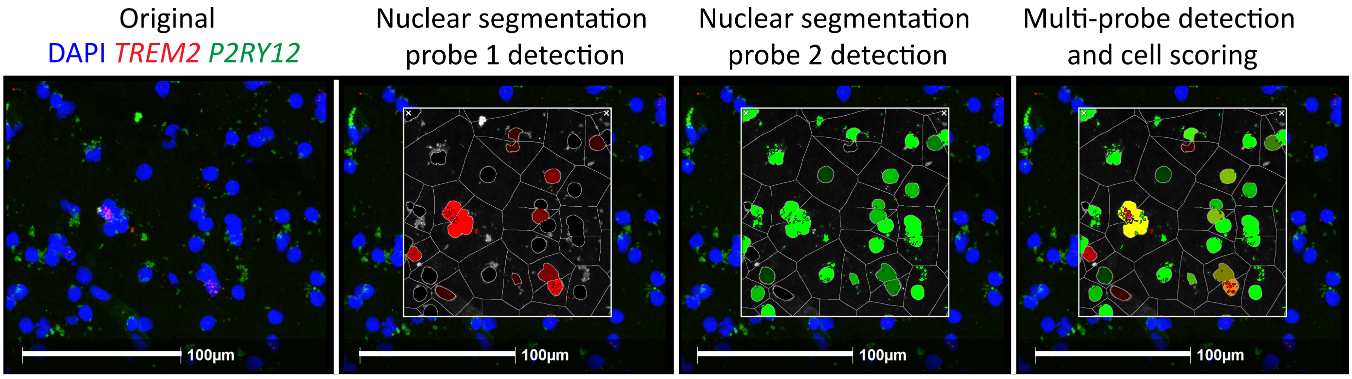

<u>Supp. Figure 1:</u> RNAscope quantification steps using HALO. Example image and graphical illustration of a 1024x1024 pixel region of interest in human brain tissue stained for *TREM2* and *P2RY12* (DAPI nuclear counterstain, C1 *TREM2* red, C2 *P2RY12* green) (left). *TREM2* expression is specific for microglia and serves as a marker to identify this cell type. Here, *P2RY12* is the gene of interest. Following nuclear segmentation, all cells on the image are separately scored for each marker on a continuous scale using the RNAscope image analysis pipeline as described in [Methods]. This will generate three populations: *TREM2+ P2RY12+* microglia, with each cell assigned a specific score for *P2RY12* expression; *TREM2+ P2RY12-* microglia; *TREM2*-cells of other lineages with varying amounts of *P2RY12*+ expression. Marker expression for each cell is measured as absolute probe counts and used to generate H-scores for downstream analysis. Coloured mark-ups for visual control of nuclear segmentation and cell classification are generated based on the analysis data (middle and right panels). Here, probe signals are indicated as bright red (probe 1, *TREM2*) or bright green (probe 2, *P2RY12*) dots on the image. White lines indicate nuclear contours and cell boundaries. Probe signals are assigned to each nucleus by proximity within a maximum cell radius of 25µm. Coloured nuclear overlays represent cell-scores as 0+ (black), and 1+ to 4+ with increasing colour intensity representing increasing counts for probe 1 (red, second panel) or probe 2 (green, third panel) per cell as described in [Methods]. Summary scores are derived for each cell on the image with graphical overlays highlighting cells with probe 1 signal only as red (*TREM2+ P2RY12-* microglia), probe 2 signal only as green (*TREM2- P2RY12+* cells of other lineages), and double positives as yellow (*TREM2+ P2RY12+* microglia). The colour shade corresponds to the total expression H-score (0-400; 0= darkest; 400=brightest).

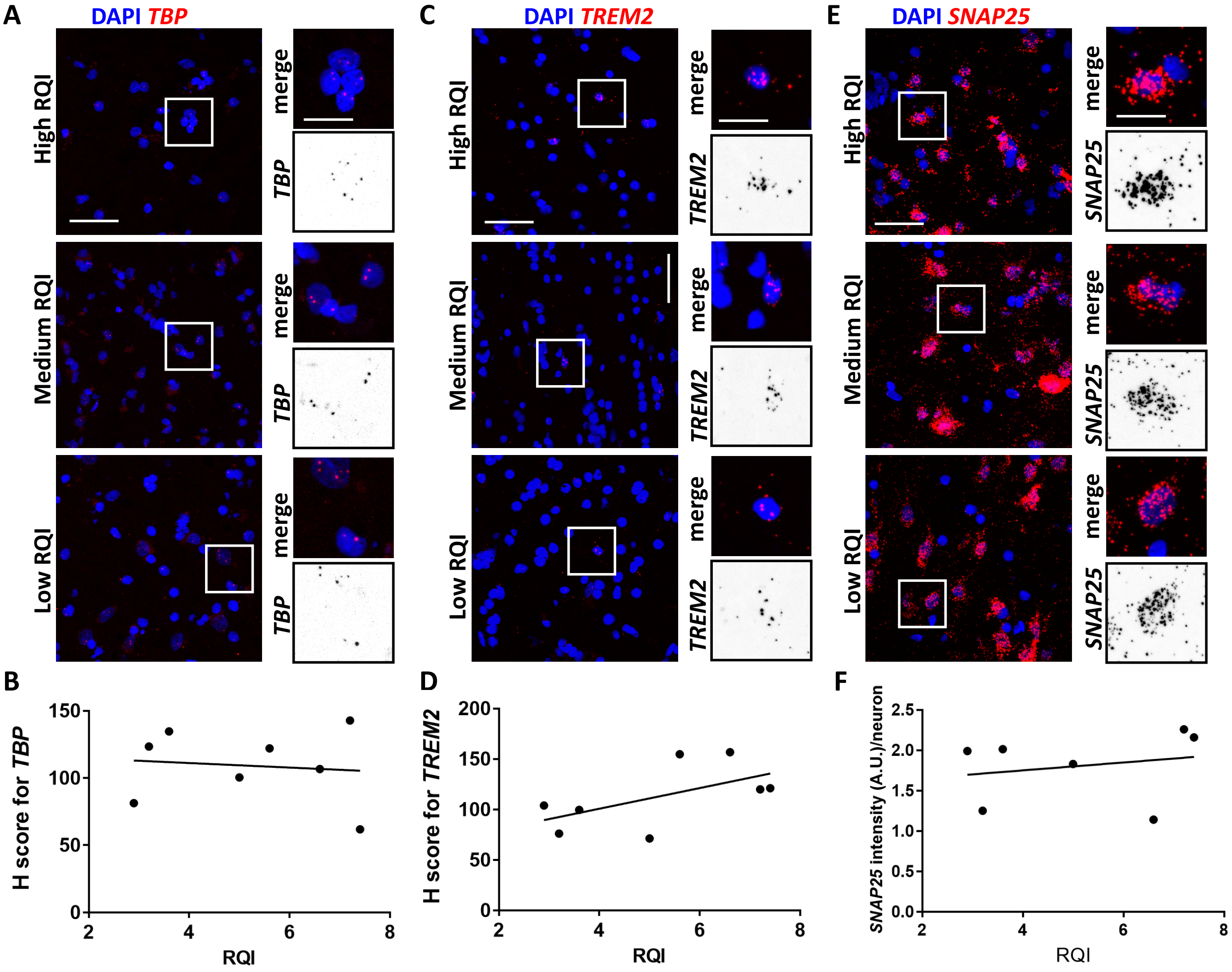

Supp. Figure 2: Low RNA quality does not affect RNAscope staining. (**A, C, E**) Confocal fluorescent RNAscope shows expression of *TBP* (A), *TREM2* (B), and *SNAP25* (E) in fresh frozen hippocampal sections with high (7.2-7.4, top panel), medium (5-6.6, middle panel) and low (2.9-3.6, bottom panel) RNA Quality Indicator (RQI). DAPI indicates nuclear staining (blue) in all confocal images. Scale bars 50 μm, zoom images 20 μm. (**B, D, F**) Quantification of the number of puncta/cell (H-score) (B, D) and of the intensity per cell (F) showed no differences in sections with different RQIs (B: *TBP* (R=-0.1112, p=0.7933); D: *TREM2* (R=0.5803 p=0.1315); F: *SNAP25* (R=0.216, p=0.642)).

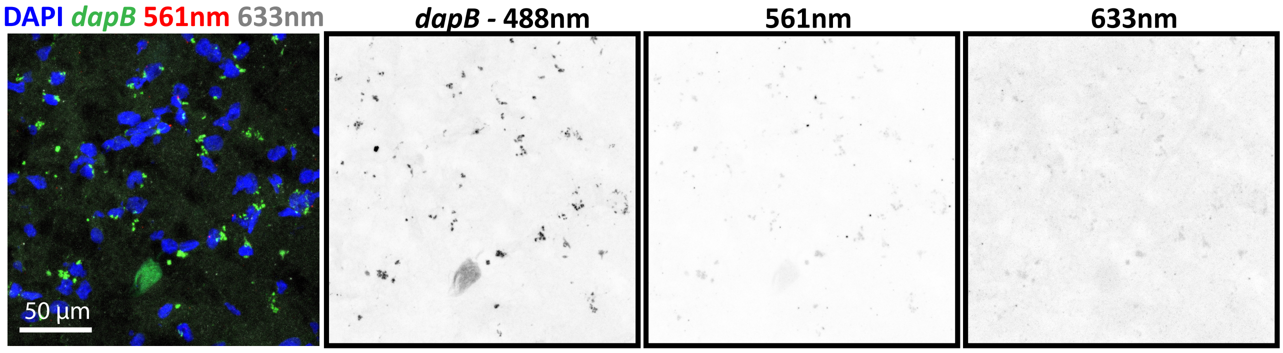

<u>Supp. Figure 3:</u> Fluorescent background levels in human neurodegenerative brain samples. RNAscope procedure was performed on human hippocampal sections. The green channel is stained for the negative control *dapB* and presents high background signal possibly due to the large protein aggregates and lipofuscin present in brain tissue from AD patients. Minimal background is detected in the other channels.

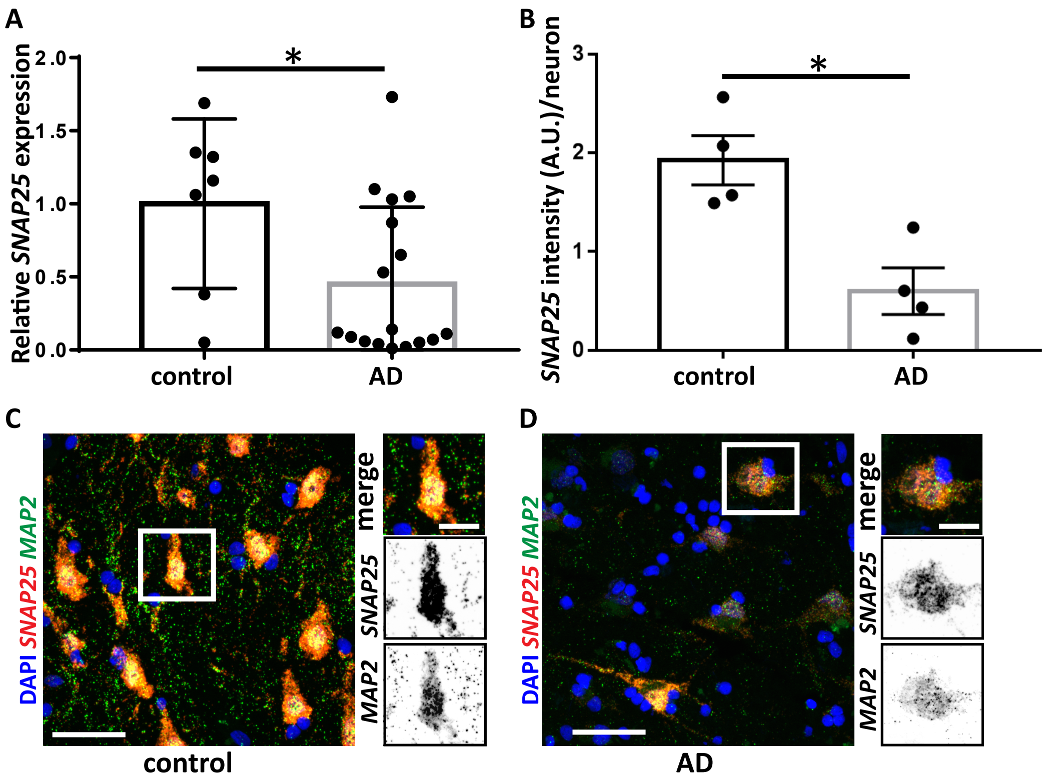

<u>Supp. Figure 4:</u> **Neuron-specific *SNAP25* mRNA levels are significantly decreased in the AD brain.** (**A**) Bulk *SNAP25* mRNA expression in human hippocampal samples. qPCR analysis showed a 55% reduction in *SNAP25* mRNA expression in AD (n=17) vs. control (n=7) samples (* indicates p=0.034 by Mann-Whitney U Test. (**B**) Quantification of *SNAP25* intensity per neuron for control (**C**; n=4) and AD (**D**; n=4) samples (* indicates p=0.0286 by Mann-Whitney U Test). (**C** and **D**) Representative confocal images of control (**C**) and AD (**D**) biopsies co-labelled with *SNAP25* and *MAP2* showing a decreased level of mRNA expression. DAPI indicates nuclear staining (blue) in all confocal images. Scale bars 50 µm, zoom images 20 µm. Graphs showing mean value ± SEM.

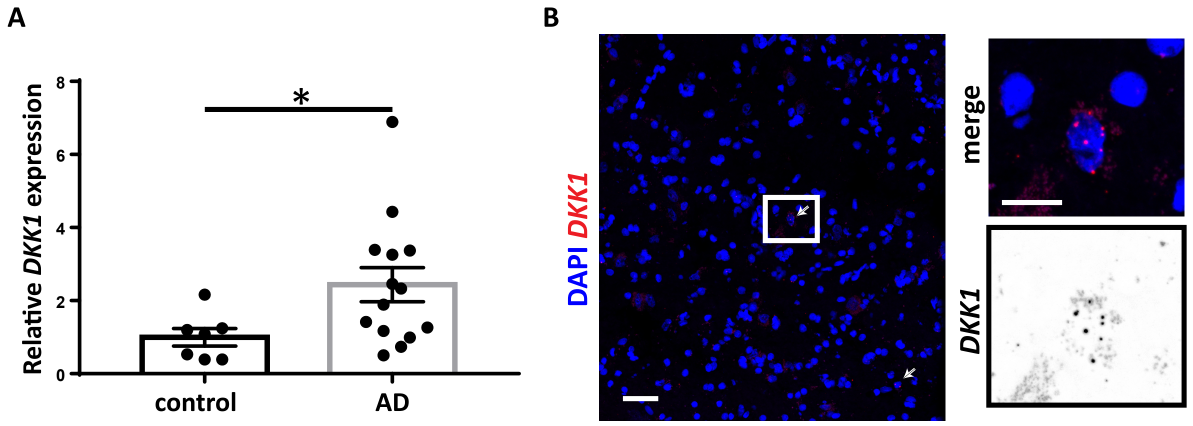

<u>Supp. Figure 5:</u> ***DKK1* expression is upregulated in AD.** (**A**) Bulk *DKK1* mRNA expression in human hippocampal samples. qPCR analysis showed a 2.44 fold increase in *DKK1* mRNA expression in AD (n=14) vs. control (n=7) samples (* indicates p=0.025 by Mann-Whitney U Test). Graphs showing mean value ± SEM. (**B**) Tile scan showing a 1350 μm^2 image, 7 times larger image than our confocal images, where only two cells are positively labelled for *DKK1*. Scale bars 50 μm, zoom images 20 μm.

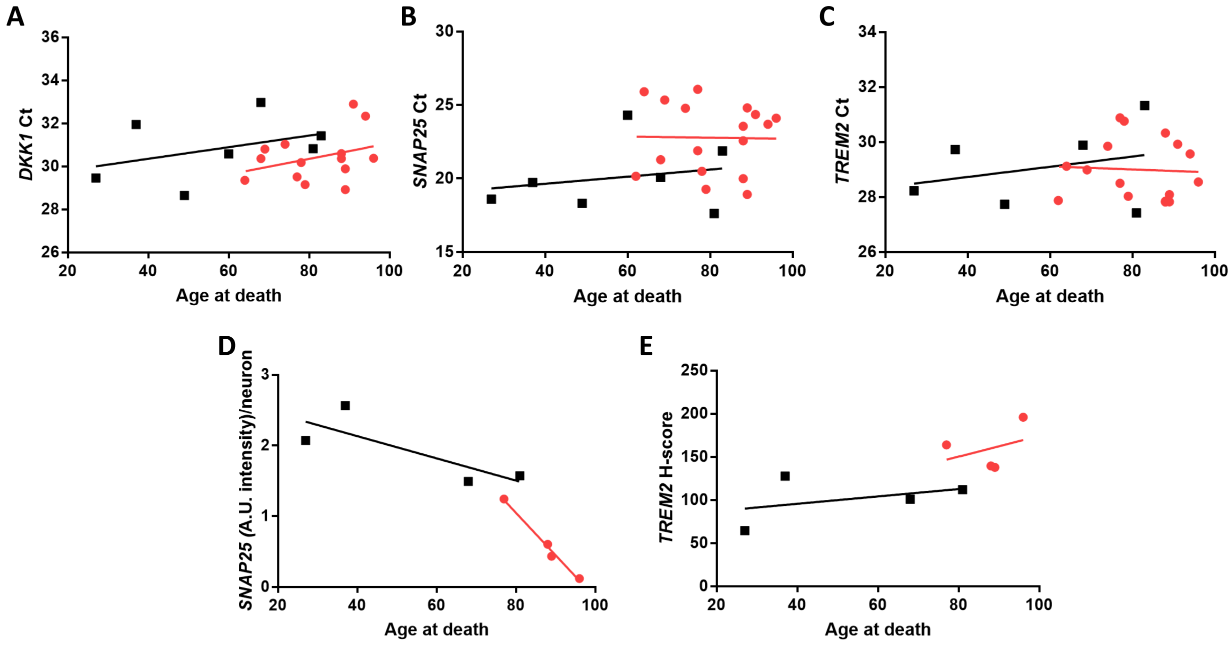

<u>Supp. Figure 6:</u> Correlation analysis between age at death and *DKK1*, *SNAP25* or *TREM2* expression. (**A-C**) *DKK1 (A)*, *SNAP25* (B) and *TREM2* (C) expression are not correlated to the age at death in controls and AD cases when analysed by qPCR (Control *DKK1*: R=0.390, p=0.382; AD *DKK1*: R=0.335, p=0.242; Control *SNAP25*: R=0.222, p=0.633; AD *SNAP25*: R-0.017, p=0.984; Control *TREM2*: R=0.288, p=0.580; AD *TREM2*: R=-0.054, p=0.843). (**D**) *SNAP25* expression is not correlated to the age at death in control samples while its expression is correlated in AD samples when analysed by smFISH (Control: R=-0.802, p=0.198; AD R=-0.994, p=0.006). (**E**) *TREM2* expression is not correlated to age at death in control or AD samples when analysed by smFISH (Control: R=0.405, p=0.595; AD: R=0.346, p=0.654). Control samples are represented by black filled squares; AD samples are represented by red filled circles.

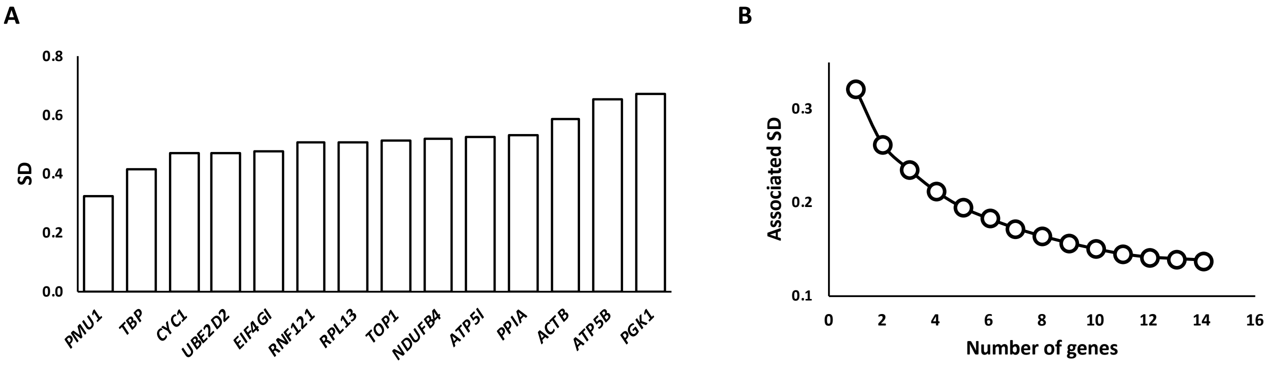

<u>**Supp. Figure 7:**</u> **Reference genes selection using GeNorm.** (**A**) Standard deviation for each possible reference gene analysed. (**B**) Associated standard deviation when pooling different possible reference genes. Based on associated SD decrease we have selected to use 4 reference genes (*PMU1*, *TBP*, *CYC1* and *UBE2D2*).

Supp. Table 1: Demographic data for human samples

		Number of samples	Gender (%)		Age at deadth (Years)				Braak Stages		RQI				PMI (h)				RQI/PMI
			Male	Female	Lower	Higher	Mean	Error	Lower	Higher	Lower	Higher	Mean	Error	Lower	Higher	Mean	Error	Pearson Correlation
**RQI impact on RNAscope**	**Controls**	**8**	**62.5**	**37.5**	**27**	**83**	**60.8**	**7.12**	**-**		**2.9**	**7.4**	**-**		**29**	**87**	**52.1**	**7.16**	**-0.046 (p = 0.622)**
**RNAscope**	**Controls**	**4**	**75.0**	**25.0**	**27**	**81**	**53.3**	**12.71**	**-**		**3.9**	**7.4**	**6.0**	**0.81**	**29**	**87**	**57.0**	**12.29**	**-0.141 (p = 0.859)**
	**AD**	**4**	**75.0**	**25.0**	**77**	**96**	**87.5**	**3.92**	**5**		**5.6**	**6.5**	**6.1**	**0.20**	**22**	**61**	**40.0**	**8.73**	**0.378 (p = 0.622)**
**qPCR**	**Controls**	**7**	**71.43**	**28.57**	**27**	**83**	**61.3**	**8.18**	**-**		**3.9**	**7.4**	**6.0**	**0.49**	**29**	**87**	**53.9**	**7.02**	**-0.056 (p = 0.905)**
	**AD**	**17**	**58.82**	**41.18**	**62**	**96**	**80.7**	**2.59**	**4**	**6**	**4.2**	**6.7**	**5.8**	**0.24**	**22**	**86**	**57.0**	**2.21**	**0.086 (p = 0.744)**

Supplementary table 1: Table containing demography data, RNA quality indicator (RQI), postmortem intervals (PMI) and Pearson correlate betwwen RQI and PMI for the different sets of samples used in each experiemnet.
RQI impcat on RNAscope: Set of data used in Figrues 1 and 2 and Supp Fig 2. **RNAscope**: dataset for Figure 3 and supp. Fig 2-3. **qPCR**: dataset for figure 3 and supp figs 4-6

Supp. Table 2: RNAscope probes

Target Gene	Species	Probe	NM accession number	REF
PPIB	Homo Sapiens	C1	NM_000942.4	313901
TBP	Homo Sapiens	C1	NM_003194.4	314291
dapB	Escherichia Coli	C1	N/A	310043
SLC1A2	Homo Sapiens	C2	NM_001166695	444721
MAP2	Homo Sapiens	C2	NM_001039538	415721
P2RY12	Homo Sapiens	C2	NM_022788	450391
SNAP25	Homo Sapiens	C1	NM_001322902	518851
TREM2	Homo Sapiens	C1	NM_001271821	420491
DKK1	Homo Sapiens	C1	NM_012242	421411

Supplementary table 2: Table containing all RNAscope probes used.

Supp. Table 3: qPCR protocol

	Cycles	Temperature	Time (s)
**Hot-start activation**	1	95°C	120
**Denaturation**	40	60°C	3
**Annealing/Extension**			30
**Ramping dissociation**	1	60-95°C	

Supplementary Table 3: Table containing the thermal cylces used for fast-qPCR

Supp. Tabel 4: Primer sequences and efficiencies

Gene	Forward			Reverse			Primer efficiency
ACTB	5'-	ACCCAGCACAATGAAGATCA	-3'	5'-	AGTACTTGCGCTCAGGAGGA	-3'	1.92
ATP5B	5'-	TCACCCAGGCTGGTTCAGA	-3'	5'-	AGTGGCCAGGGTAGGCTGAT	-3'	1.94
ATP5I	5'-	GGCAGAAGAGGAGAGGAGGA	-3'	5'-	GTCGCAGGGTCACTCACTTT	-3'	1.97
B2M	5'-	ACTGAATTCACCCCCACTGA	-3'	5'-	CCTCCATGATGCTGCTTACA	-3'	2.04
CYC1	5'-	CACGGAGGATGAAGCTAAGG	-3'	5'-	GCATGAACATCTCCCCATCT	-3'	1.97
DKK1	5'-	CCTTGGATGGGTATTCCAGA	-3'	5'-	CCTGAGGCACAGTCTGATGA	-3'	1.97
EIF4A2	5'-	AATTCCGGTCAGGGTCAAGTC	-3'	5'-	GCCACACCTTTCCTCCCAAA	-3'	1.99
EIF4G1	5'-	CTCCAGGCCCTTGTAGTGAC	-3'	5'-	CATCCTCCTTCACCACGTCC	-3'	1.95
ELFN2	5'-	TCTCATTGCTACTGCTGCCG	-3'	5'-	TGCACCTGGTTCCAGATGTC	-3'	2.00
GSK3B	5'-	ACAACAGTGGTGGCAACTCC	-3'	5'-	TTCTTGATGGCGACCAGTTCT	-3'	1.95
GUSB	5'-	GGTTGGAGAGCTCATTTGGA	-3'	5'-	CTCTCGTCGGTGACTGTTCA	-3'	1.97
MCU	5'-	GGCTCCCCTGGAAAAGGTAC	-3'	5'-	CCACCCCATAGCACCAAAGT	-3'	1.96
NDUFB4	5'-	GAGCCCAGCTGAAACGAGAG	-3'	5'-	TCTTGCATAGGCCCAACGAA	-3'	1.92
PGK1	5'-	CAGTTTGGAGCTCCTGGAAG	-3'	5'-	CACAGGAACTAAAAGGCAGGA	-3'	1.93
PMU1	5'-	AGTGGGGGACTAGGCGTTAG	-3'	5'-	GTTTTCATCACTGTCTGCATCC	-3'	1.93
PPIA	5'-	TCCTGGCATCTTGTCCATG	-3'	5'-	CCATCCAACCACTCAGTCTTG	-3'	1.98
PPIB	5'-	TGGCCTTAGCTACAGGAGAGA	-3'	5'-	TCTCCCCTGGTGAAGTCTCC	-3'	1.93
PRPL13	5'-	CCTGGAGGAGAAGAGGAAAGAGA	-3'	5'-	TTGAGGACCTCTGTGTATTTGTCAA	-3'	1.90
RNF121	5'-	TTGTTACCTTCCGAGCCACC	-3'	5'-	GCCATGTAGCCAACAATGCC	-3'	1.93
RPS18	5'-	CGTCACTTCCGCTCTCTCTT	-3'	5'-	TCGCAAAATATGCTGGAACTT	-3'	1.92
SNAP25	5'-	CTGGAAAGCACCCGTCGTAT	-3'	5'-	TCCCTTCCTCAATGCGTTCC	-3'	1.94
TBP	5'-	GCCCGAAACGCCGAATATAA	-3'	5'-	AATCAGTGCCGTGGTTCGTG	-3'	1.95
TOP1	5'-	GGCGAGTGAATCTAAGGATAATGAA	-3'	5'-	TGGATATCTTAAAGGGTACAGCGAA	-3'	1.93
TREM2	5'-	CACAGCATCTCCAGGAGCC	-3'	5'-	TGAGAAAGATGCAGGCCAG	-3'	1.91
UBC	5'-	CACTTGGTCCTGCGCTTGA	-3'	5'-	TTATTGGGAATGCAACAACTTTAT	-3'	1.93
UBE2D2	5'-	CAGCACAGTGTTCAGCAGGT	-3'	5'-	TCATTTGGCCCCATTATTGT	-3'	1.97

Supplementary Table 4: Table containing forward and reverse primer for all genes analyesed and their eficiency.

Supp. Table 5: GeNorm results for intra- and inter-groups analysis

Gene	Intra-groups		Inter-groups	
	Control	AD	Control	AD
ACTB	0.041	0.467	-0.134	0.134
ATP5B	0.224	0.473	-0.060	0.060
B2M	**1.583**	**0.705**	**0.260**	**-0.260**
CYC1	0.052	0.268	-0.016	0.016
EIF4A2	0.480	0.305	**-0.280**	**0.280**
GSK3B	0.139	0.194	**-0.276**	**0.276**
GUSB	**0.531**	0.201	**0.271**	**-0.271**
PGK1	0.325	0.462	-0.065	0.065
PPIA	0.032	0.405	-0.147	0.147
PMU1	0.034	0.091	0.074	-0.074
RPL13	0.405	0.138	0.186	-0.186
RPS18	0.046	0.287	**-0.221**	**0.221**
TBP	0.040	0.166	-0.062	0.062
TOP1	0.066	0.288	0.093	-0.093
UBC	0.215	0.456	**0.415**	**-0.415**
UBE2D2	0.087	0.222	-0.034	0.034
ATP5I	0.103	0.390	0.032	-0.032
EIF4GI	0.015	0.351	0.152	-0.152
ELFN2	**1.024**	**2.510**	**-0.508**	**0.508**
MCU	0.284	**0.619**	0.183	-0.183
NDUFB4	0.018	0.432	0.041	-0.041
RNF121	0.005	0.415	0.097	-0.097

Supplementary table 5: Table containing GeNomr results for intra-and inter-groups analysis. Red marks standard deviations over the cut-off applied: 0.5 for intra-group and ± 0.2 for inter-group
